# Galactosylated Prodrugs: A Strategy to Improve the Profile of Nonsteroidal Anti-Inflammatory Drugs

**DOI:** 10.3390/ph15050552

**Published:** 2022-04-29

**Authors:** Federica Sodano, Claudia Cristiano, Barbara Rolando, Elisabetta Marini, Loretta Lazzarato, Mariarosaria Cuozzo, Stefania Albrizio, Roberto Russo, Maria Grazia Rimoli

**Affiliations:** 1 Department of Pharmacy, “Federico II” University of Naples, 80131 Naples, Italy; claudia.cristiano@unina.it (C.C.); mariarosaria.cuozzo@unina.it (M.C.); salbrizi@unina.it (S.A.); roberto.russo@unina.it (R.R.); rimoli@unina.it (M.G.R.); 2 Department of Drug Science and Technology, University of Turin, 10125 Turin, Italy; barbara.rolando@unito.it (B.R.); elisabetta.marini@unito.it (E.M.); loretta.lazzarato@unito.it (L.L.)

**Keywords:** galactose, prodrug approach, NSAIDs, physicochemical characterization, stability, serum behavior, intestinal permeation, pain, inflammation, ulcerogenicity

## Abstract

Carbohydrates are one of the most abundant and important classes of biomolecules. The variety in their structures makes them valuable carriers that can improve the pharmaceutical phase, pharmacokinetics and pharmacodynamics of well-known drugs. D-galactose is a simple, naturally occurring monosaccharide sugar that has been extensively studied for use as a carrier and has proven to be valuable in this role. With the aim of validating the galactose-prodrug approach, we have investigated the galactosylated prodrugs ibuprofen, ketoprofen, flurbiprofen and indomethacin, which we have named IbuGAL, OkyGAL, FluGAL and IndoGAL, respectively. Their physicochemical profiles in terms of lipophilicity, solubility and chemical stability have been evaluated at different physiological pH values, as have human serum stability and serum protein binding. Ex vivo intestinal permeation experiments were performed to provide preliminary insights into the oral bioavailability of the galactosylated prodrugs. Finally, their anti-inflammatory, analgesic and ulcerogenic activities were investigated in vivo in mice after oral treatment. The present results, taken together with those of previous studies, undoubtedly validate the galactosylated prodrug strategy as a problem-solving technique that can overcome the disadvantages of NSAIDs.

## 1. Introduction

Prodrug design is a well-known strategy that consists of chemically modifying the structure of a drug, which is called the “parent drug”. This approach allows the physicochemical and pharmacological properties of a parent drug to be optimized, resulting in an improvement in its pharmacokinetic characteristics and a decrease in its toxicity [1,2,3]. Prodrugs mainly fall into two categories: bioprecursors and carrier prodrugs. In the former, the molecule acts as a substrate for enzymes, which then release the active drug [4,5], whereas, in the latter, the parent drug is linked through enzymatically or chemically cleavable groups to a molecule that acts as a carrier [4]. The carrier can also be a polymer, and, in this case, they are known as macromolecular prodrugs [6,7]. Another class bears the name mutual prodrugs; here, two active compounds are linked, with each acting as a transporter of the other [4,5]. Carbohydrates are an example of successful carriers.

Carbohydrates are one of the most important and abundant classes of biomolecules [8], as they are major components of genetic material and are the main vital source of energy [9]. They play essential roles in cellular and intracellular interactions, thanks to their action as signaling molecules and their presence on cell-surface receptors [10]. These functions explain the potential of carbohydrates for use as pharmaceutical and diagnostic agents and thus reinforce the importance of the topic covered in this Special Issue.

Medicinal chemists are encouraged to design and adopt strategies to synthesize carbohydrate-based drugs [9,11]. The variety present in carbohydrate structures makes these molecules a valuable tool for the development of biologically active glycoconjugates and carrier prodrugs [12]. In particular, the presence of the carbohydrate moiety grants carrier prodrugs good hydrophilic–lipophilic balance, resulting in decreased toxicity and increased bioavailability [13].

D-galactose is a simple, naturally occurring monosaccharide sugar that has been extensively studied for use as a carrier in the prodrug approach. The use of galactosylated prodrugs has proven to be an excellent strategy, particularly when parent drugs are likely to fail therapeutically due to a lack of site-of-action specificity, a poor toxicological profile and chemical instability. It has been shown, in many studies, that the binding of a parent drug to D-galactose increases its selectivity of action and reduces its side effects. In other cases, the presence of the sugar structure ensures an improvement in the solubility and cell permeability of the parent drug, and thus in its pharmacokinetic profile [14,15], while keeping its pharmacodynamic characteristics intact [16].

Nonsteroidal anti-inflammatory drugs (NSAIDs) are among the most widely used therapeutic agents worldwide [17], making up approximately 5% of all prescribed drugs [18,19]. Despite their high therapeutic potential, long-term exposure to NSAIDs causes serious cardiovascular events, including myocardial infarction and stroke, and gastrointestinal damage, such as bleeding, ulceration and perforation, which can be fatal, as well as kidney injury, hepatotoxicity and other, more minor ailments [20,21,22,23].

There are two factors that contribute to both mild and severe gastrointestinal adverse events: firstly, the direct irritating contact of the free carboxylic function of NSAIDs with the gastrointestinal mucosa (the ion-trapping phenomenon); and secondly, the blockage of prostaglandin biosynthesis, which has more indirect effects. These adversities have severely limited the clinical use of NSAIDs and highlight the unmet need for new therapeutic strategies that aim to make their prolonged usage safer [24].

Promising results have been obtained with the prodrug approach when using D-galactose as a carrier for NSAIDs. In our previous studies, the conjugation of a galactose molecule with ketorolac (Ketogal) [25], paracetamol (PARgal) [26] and aceclofenac (ACEgal) [27] improved their physicochemical, toxicological, pharmacokinetic and pharmacodynamic profiles, resulting in remarkable safety profiles. In particular, the prolonged pharmacological action of the aforementioned prodrugs, in comparison with their parent drugs, would allow their daily intake to be reduced, while also decreasing side effects. At the same time, it may also be possible to reduce or eliminate the use of drugs that are commonly prescribed with NSAIDs, such as proton pump inhibitors (PPI), with consequent benefits in terms of health and public spending.

**Figure 1:**
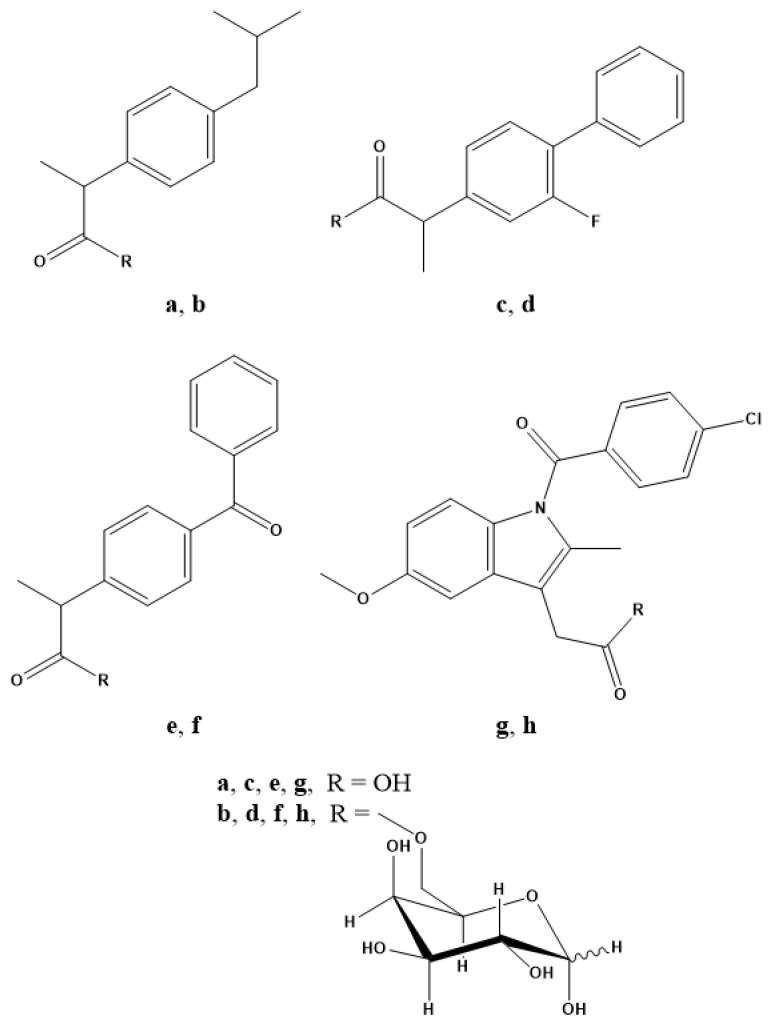
Chemical structures of (**a**) ibuprofen, (**b**) IbuGAL, (**c**) flurbiprofen, (**d**) FluGAL, (**e**) ketoprofen, (**f**) OkyGAL, (**g**) indomethacin and (**h**) IndoGAL.

In light of the results obtained and with the aim of validating the galactose-prodrug approach, we have investigated the galactosylated prodrugs of ibuprofen, ketoprofen, flurbiprofen and indomethacin, which we have named IbuGAL, OkyGAL, FluGAL and IndoGAL, respectively, the syntheses of which have been described previously and the structures of which are reported in Figure 1 [28]. In this work, we evaluate their physicochemical profiles in terms of lipophilicity, solubility and chemical stability at different physiological pH values (7.4 and 1.2). The serum behavior of IbuGAL, OkyGAL, FluGAL and IndoGAL was investigated by measuring human serum stability and serum protein binding. Ex vivo intestinal permeation experiments were carried out to provide preliminary insights into the oral bioavailability of these galactosylated prodrugs. Finally, the in vivo pharmacological and toxicological profiles of galactosylated prodrugs were evaluated. Specifically, anti-inflammatory (carrageenan-induced paw edema test), analgesic (acetic acid-induced writhing test) and ulcerogenic activity were investigated in mice after oral treatment.

## 2. Results

### 2.1. Physicochemical Characterization

#### 2.1.1. Lipophilicity

Lipophilicity is the key physicochemical parameter that influences solubility and various other aspects of the pharmacokinetic and pharmacodynamic profile of drugs. The hydrophilic-lipophilic behavior of galactosylated prodrugs and parent drugs was investigated by measuring the distribution coefficients (log D^pH^) in *n*-octanol-phosphate buffered saline (PBS, pH 7.4) using the shake-flask technique. The distribution coefficients of the galactosylated prodrugs were found to be pH-independent, which reflects the absence of ionizable groups, meaning that the log D^7.4^ values were superimposable on the calculated log *p*-values (ClogP), as shown in Table 1. A log D value’s proximity to two (as in the case of galactosylated prodrugs) suggests optimal pharmacokinetic/pharmacodynamic behavior. Ibuprofen, ketoprofen, flurbiprofen and indomethacin, which are broadly ionized at pH 7.4, exhibited lipophilicity at about one logarithmic unit lower than the related prodrugs.

#### 2.1.2. Solubility Assay

The solubilities of the prodrugs and parent drugs were evaluated at 25 °C in water, simulated gastric fluid (SGF without pepsin, pH 1.2) and PBS (pH 7.4), which are the media used for chemical stability analyses. The solubilities were quantified using RP-HPLC. As expected, the galactosylated derivatives were more soluble than the relative parent drugs in both water and SGF. Conversely, the solubility of the parent drugs at pH 7.4 increased significantly due to their ionization (Table 1).

#### 2.1.3. Stability in SGF and PBS

The chemical stability of galactosylated prodrugs was evaluated over 48 h at 37 °C in SGF and PBS in order to simulate the gastric and physiological environments, respectively. The concentrations of the galactosylated prodrugs and the parent drugs derived from the prodrugs were quantified using RP-HPLC (Table 2). SGF was the experimental environment in which the prodrugs displayed the highest stability; the release of the parent drugs from the relative prodrugs after 24 h of incubation in SGF was lower than 4% and therefore considered negligible. The galactosylated prodrugs were shown to be stable even at physiological pH; the percentage of parent drugs released after 24 h of incubation in PBS was about 10%.

#### 2.1.4. Stability in Human Serum

Stability was also evaluated in human serum at 37 °C over 48 h (Table 2). In this esterase-rich medium, the degradation of galactosylated prodrugs into their parent drugs was more evident. For IbuGAL and FluGAL, the half-life was close to 24 h (ca 50% degradation), whereas stability increased slightly for OkyGAL (ca 30% degradation in 24 h). However, IndoGAL was the most stable compound under these conditions (ca 20% degradation in 24 h). The parent drugs were the only degradation products of the galactosylated prodrugs that were observed using RP-HPLC in these stability studies.

#### 2.1.5. Serum Protein Binding

The serum protein binding properties of the galactosylated prodrugs and parent drugs were investigated using ultrafiltration. The compounds were incubated in human serum, and after 1 h, the concentrations of the compounds in the ultrafiltrate and the original serum were determined by means of RP-HPLC, and used to calculate the binding percentages, which are reported in Table 2. The prodrugs, with the exception of IndoGAL, showed slightly lower percentages of binding to serum proteins than their relative parent drugs, although they maintained binding percentages above 95% (Table 2).

### 2.2. Ex Vivo Absorption Evaluation

The permeability of the galactosylated prodrugs and the parent drugs through the rat small intestine was assessed ex vivo using the non-everted intestinal sac method. Despite some shortcomings (e.g., the interruption of normal blood flow, lack of a nervous system) this test is widely used to investigate the passive absorption of molecules [29] and can provide further data when equipped with a mucus layer, which includes proteins of drug transport and metabolism [30].

A suspension of the tested compounds (5 mg/Kg in PBS + methocel 3%) was syringed into intestinal sacs that consisted of different segments of rat small intestine (duodenum, jejunum, ileum). The compound-enriched tissues were incubated at 37 °C for 2 h. Samples (0.5 mL) were withdrawn every 15 min from the serosal side and the amount of analytes was determined via RP-HPLC. The results are collected in Table 3.

An analysis of the solutions withdrawn during the incubation of intestinal sacs filled with the parent drugs showed very heterogeneous behavior, with ibuprofen demonstrating high absorption (fraction absorbed, F_a_, ranging from 0.84 to 0.98), flurbiprofen and ketoprofen demonstrating intermediate absorption (F_a_ ranging from 0.57 to 0.69 and from 0.46 to 0.53, respectively) and indomethacin demonstrating very low absorption (F_a_ ranging from 0.040 to 0.094). The three different intestinal segments showed no significant differences in absorption.

**Figure 2:**
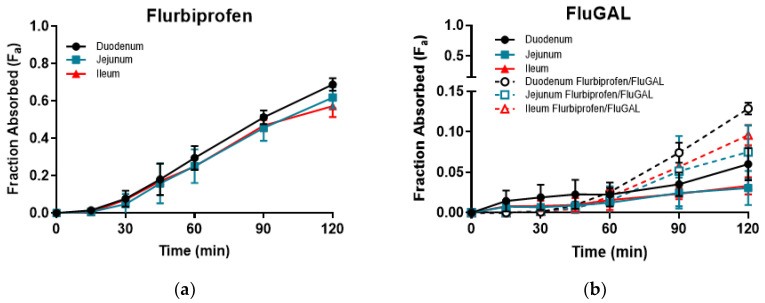
The time course of ex vivo fraction absorbed (F_a_) of flurbiprofen (**a**) and FluGAL (**b**) in the three intestinal tracts.

On the other hand, all of the galactosylated prodrugs showed similar profiles, and intestinal permeations that were significantly lower than those of their relative NSAIDs. Indeed, an analysis of the samples withdrawn revealed that very low amounts of IbuGAL, OkyGAL, FluGAL and their parent drugs had been released. IndoGAL and, consequently, indomethacin could not be evaluated in any of the three intestinal tracts (Table 3). An example of the time course of F_a_ in the three intestinal tracts is reported in Figure 2.

**Figure 3:**
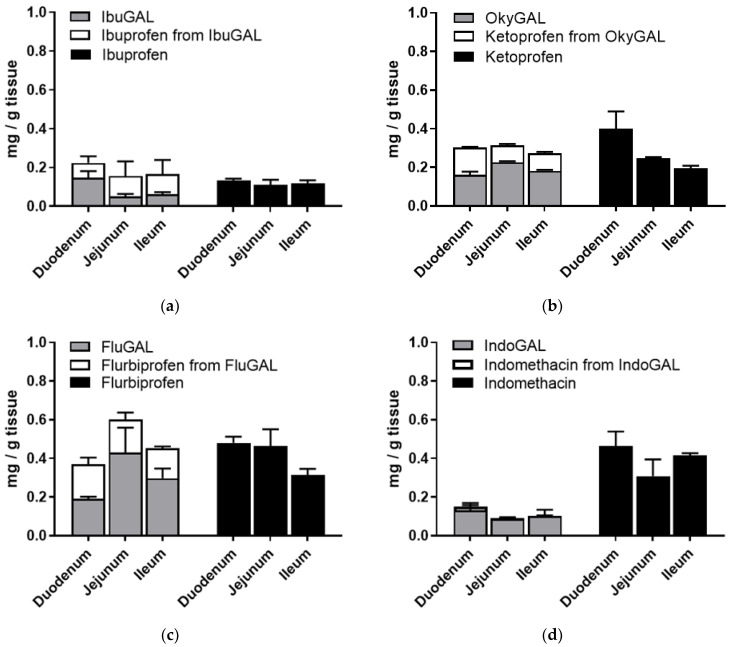
(**a–d**) Ex vivo intestinal permeation. Tissue accumulation of parent drugs (**a**) ibuprofen, (**b**) ketoprofen, (**c**) flurbiprofen and (**d**) indomethacin and the galactosylated prodrugs (**a**) IbuGAL, (**b**) OkyGAL, (**c**) FluGAL and (**d**) IndoGAL. Non-everted intestinal sacs were filled with a suspension of the compounds (5 mg/Kg, PBS + methocel 3%) and incubated in oxygenated Krebs–Ringer buffer at 37 °C with smooth shaking. At the end of the incubation period, the tissues were washed with fresh buffer, homogenized in ethanol and centrifuged and the supernatant was dried. The concentrations of the parent drugs and the galactosylated prodrugs and the parent drugs derived from the prodrugs were quantified using RP-HPLC. Tests were performed in triplicate on intestinal segments from three different rats.

In order to verify the presence of the galactosylated compounds in the intestinal tissues, the intestinal sacs were opened at the end of the incubation period, washed with fresh buffer and homogenized in ethanol and the supernatant was dried. The concentrations of the galactosylated prodrugs and the prodrug-derived parent drugs were quantified using RP-HPLC (Figure 3a–d).

As shown in Figure 3a–d, an analysis of the tissue accumulation highlighted the presence of significant amounts of the galactosylated compounds and the derived parent drugs. In particular, the total amount of prodrug plus derived parent drug was equal to the tissue accumulation of the relative NSAID alone.

### 2.3. In Vivo Studies

#### 2.3.1. Effect of Parent Drugs and Galactosylated Prodrugs on Paw Edema in Mice

We tested the anti-inflammatory response of the parent drugs and their relative galactosylated prodrugs by subcutaneously injecting λ-carrageenan (1% (*w*/*v*) in sterile saline, 20 μL) into the hind paws of mice. Evaluation continued for 96 h after the carrageenan challenge.

**Figure 4:**
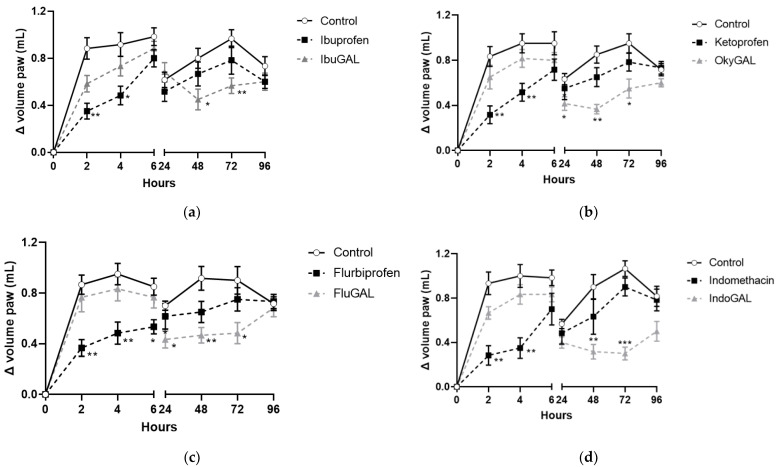
(**a**–**d**). Effect of the parent drugs and galactosylated prodrugs on carrageenan-induced hyperalgesia. Effect of oral administration of the parent drugs (**a**) ibuprofen, (**b**) indomethacin, (**c**) ketoprofen and (**d**) flurbiprofen and the galactosylated prodrugs (**a**) IbuGAL, (**b**) IndoGAL, (**c**) OkyGAL and (**d**) FluGAL on carrageenan-induced hyperalgesia in mouse paws evaluated at 2, 4, 6, 24, 48, 72 and 96 h after the carrageenan challenge. White circles represent control mice treated with vehicle. Black squares represent mice treated with the parent drugs (ibuprofen, indomethacin, ketoprofen, and flurbiprofen) and gray triangles represent mice treated with the galactosylated prodrugs (IbuGAL, IndoGAL, OkyGAL and FluGAL). The increase in paw volume was evaluated and is expressed as the difference in paw volume measured at each time point and the basal paw volume measured immediately before carrageenan injection. Data are expressed as means ± S.E.M. of 6 animals. *** *p* < 0.001, ** *p* < 0.01 and * *p* < 0.5 vs. Control.

Ibuprofen, indomethacin, ketoprofen and flurbiprofen were administered orally at a dose of 10 mg/kg (Figure 4a–d, respectively) and they inhibited paw edema formation during the first phase (0–6 h). The anti-inflammatory activity was significant at 2 and 4 h after carrageenan injection for ibuprofen, indomethacin and ketoprofen and up to 6 h for flurbiprofen. The antihyperalgesic effect completely disappeared after 24 h.

By contrast, equimolecular doses of IbuGAL (17.8 mg/kg), IndoGAL (14.5 mg/kg), OkyGAL (16.4 mg/kg) and FluGAL (16.7 mg/kg) did not change the carrageenan-induced hyperalgesia during the first phase of the test, but displayed significant anti-inflammatory activity during the second phase (24–96 h). In particular, IbuGAL and IndoGAL (Figure 4a,b, respectively) showed an anti-inflammatory effect from 48 to 72 h following carrageenan administration, whereas OkyGAL and FluGAL significantly reduced paw edema from 24 to 72 h (Figure 4c,d, respectively).

#### 2.3.2. Effect of Parent Drugs and Galactosylated Prodrugs on the Acetic-Acid-Induced Writhing Test in Mice

**Figure 5:**
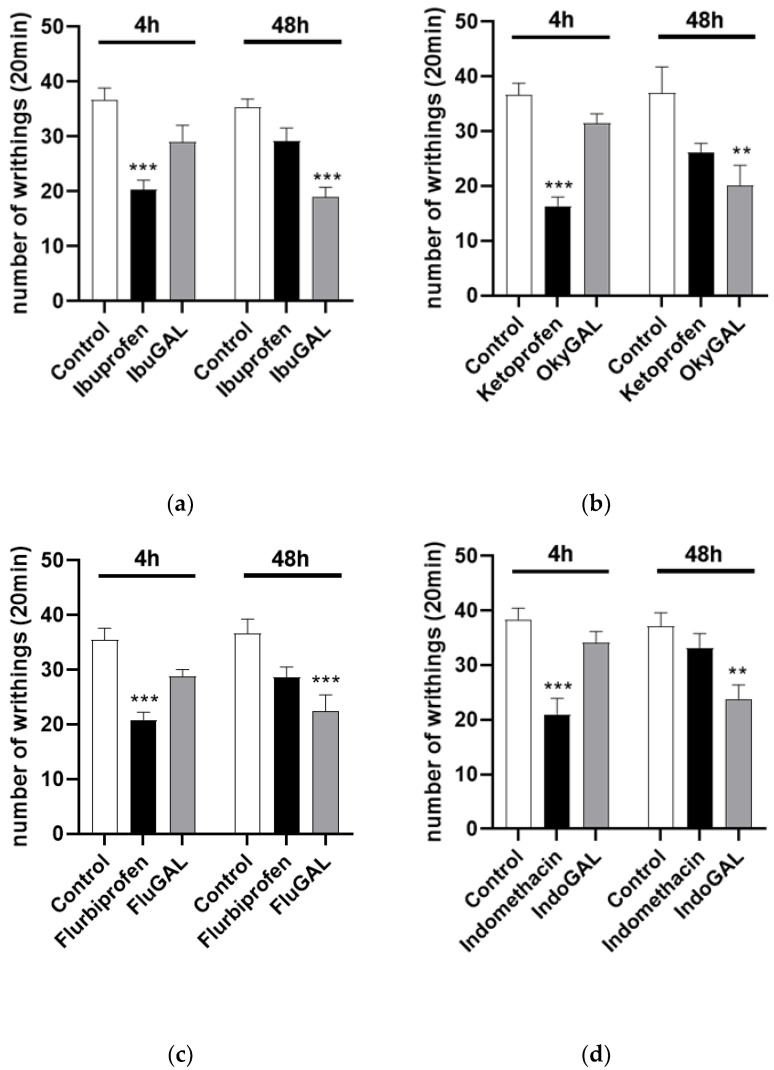
(**a**–**d**). Total number of writhing episodes within 20 min after acetic acid injection. Effect of oral administration of the parent drugs (**a**) ibuprofen, (**b**) indomethacin, (**c**) ketoprofen and (**d**) flurbiprofen and the galactosylated prodrugs (**a**) IbuGAL, (**b**) IndoGAL, (**c**) OkyGAL and (**d**) FluGAL at 4 and 48 h after administration of 0.6% acetic acid (10 mL/kg). Data are expressed as means ± S.E.M. of 6 animals. *** *p* < 0.001, ** *p* < 0.01 vs. Control.

The number of acetic-acid-induced abdominal stretches can be evaluated in Figure 3. Control mice received an intraperitoneal injection of 0.6% acetic acid (10 mL/kg). Oral treatment was started 1 h before acetic acid administration. Ibuprofen, indomethacin, ketoprofen and flurbiprofen significantly reduced the number of writhings after 4 h, at a dose of 10 mg/kg (Figure 5a–d, respectively), compared to the control group. Conversely, IbuGAL (17.8 mg/kg), IndoGAL (14.5 mg/kg), OkyGAL (16.4 mg/kg) and FluGAL (16.7 mg/kg) (Figure 4a–d, respectively) resulted in a significant decrease, compared to the control group, in writhing responses after 48 h.

#### 2.3.3. Effect of Parent Drugs and Galactosylated Prodrugs on Ulcerogenic Activity

The study of ulcerogenic activity indicated that, as expected, the parent drugs (ibuprofen, indomethacin, ketoprofen and flurbiprofen), which have free acid functions, showed significantly higher ulcerogenic properties at 4 h and only indomethacin showed these increased properties at 48 h (values between 1.5–3) after oral treatment, compared with the control. However, the galactosylated prodrugs (IbuGAL, IndoGAL, OkyGAL and FluGAL) displayed less ulceration than the parent drugs at the same experimental times (values between 0–1.5). The results are reported in Table 4.

## 3. Discussion

The task of a drug is to perform only its biological activity at the target site, at the lowest concentration, while avoiding side effects [31]. Several approaches have been developed to achieve this goal, including the preparation of carbohydrate-based prodrugs. The conjugation of the parent drug to a carbohydrate, such as D-galactose, is a recent strategy that still requires validation [15,32]. We applied the galactosylated prodrug strategy to a number of NSAIDs. The first successful example of this approach was Ketogal [25], with ACEgal and PARgal then following [26,27]. In each study, the galactosylated prodrug showed marked improvements, compared with its parent drug, in some or all of its physicochemical, pharmacokinetic, pharmacodynamic and toxicological parameters.

In order to confirm the suitability of D-galactose as a carrier in the development of analgesic and anti-inflammatory treatments, we examined a number of NSAIDs that had already been subjected to the galactosylated prodrug strategy in terms of design and synthesis. IbuGAL, OkyGAL, FluGAL and IndoGAL (Figure 1) were synthesized via a green method using room-temperature ionic liquids, as reported in our previous work [28].

In evaluating the physicochemical profiles of the galactosylated prodrugs, it was seen that conjugation with a D-galactose molecule improved the solubility of the starting drug (Table 1). Specifically, IbuGAL, OkyGAL and FluGAL were more soluble than ibuprofen, ketoprofen and flurbiprofen, respectively, in both water and SGF. In PBS, of course, the three starting drugs were more soluble as they are deprotonated at pH = 7.4. Surprisingly, IndoGAL was as soluble as indomethacin in water, despite the increase in the number of sugar-component hydroxyl groups. No great differences between IndoGAL and indomethacin were observed in SGF, whereas the solubility of IndoGAL at pH 7.4 was lower than that of its parent drug, as observed for the other galactosylated derivatives.

The solubility trends of the prodrugs and parent drugs in PBS reflect the values of the distribution coefficients (log D^7.4^), which were measured under the same conditions (Table 1). The decrease in prodrug solubility at pH 7.4, compared to the parent drugs, is in agreement with the increase in their log D by one logarithmic unit, again compared to the parent drugs. The large difference between ClogP and log D^7.4^ in the parent drugs is in accordance with their significant ionization at physiological pH (Table 1).

The galactosylated prodrug strategy ensures that the chemical stability of NSAIDs is maintained in the relative prodrugs. The percentage of unmodified IbuGAL, OkyGAL, FluGAL and IndoGAL remained greater than 97% in SGF and 81% in PBS after 24 h of incubation (see Table 2).

The galactosylated prodrugs also demonstrated stability in human serum. In fact, IndoGAL proved to be the most stable compound, followed by OkyGAL, whereas FluGAL and IbuGAL showed a half-life of around 24 h. With regards to serum protein binding, the binding percentage of ibuprofen, ketoprofen, flurbiprofen and indomethacin was 99.98%, which is in agreement with the literature (Table 2) [33]. The galactosylated prodrug strategy led to a decrease in the binding of NSAIDs to serum proteins and thus to increased free-prodrug availability in circulation.

Absorption is a primary goal in drug development and medicinal chemistry, as a drug must be absorbed before any medicinal effect can take place. In addition, the pharmacokinetic profile of a drug can be easily and significantly changed by adjusting the factors that influence absorption. In our case, the conjugation of NSAIDs to galactose led to significant changes in the absorption profile (Table 3). In fact, in the ex vivo intestinal permeation study, differences were found in the degree of absorption of the galactosylated prodrugs compared to the parent drugs, over the two-hour course of the test. This aspect may be considered disadvantageous, but, generally, the absorption and pharmacological activity of a prodrug must be delayed and prolonged over time. The permeability of IbuGAL, OkyGAL, FluGAL and IndoGAL through the rat small intestine was lower than that of the starting drugs, suggesting that the galactosylated prodrugs exhibited lower in vivo oral absorption over the first two hours. However, the small fraction that is orally absorbed would nevertheless be able to play its pharmacological role, as shown by the results of the anti-inflammatory and analgesic activity tests. The reduced binding to serum protein and the high half-life in human serum of the galactosylated prodrugs resulted in improved drug bioavailability.

No change in anti-inflammatory (Figure 3) and analgesic (Figure 4) potential was found when comparing the pharmacological profiles of the galactosylated prodrugs and their respective NSAIDs. Despite being absorbed less, IndoGAL had the same efficacy of action as indomethacin.

The anti-inflammatory activity of IbuGAL, OkyGAL, FluGAL and IndoGAL was found to be delayed and prolonged over time when assessed using the carrageenan-induced edema test. Specifically, the anti-inflammatory action of NSAIDs began 2 h after oral treatment and lasted up to 4 h, except for that of flurbiprofen, which persisted up to 6 h [34,35,36,37]. IbuGAL and IndoGAL exhibited their activity 48 h after oral administration and this lasted up to 72 h, whereas the activity of FluGAL and OkyGAL started after 24 h. The development of NSAIDs with prolonged and time-delayed activity may be advantageous as these drugs are commonly prescribed for chronic inflammatory diseases. The repeated use of NSAIDs could therefore be completely subverted by the administration of a single dose of their galactosylated analogues.

The analgesic activity of galactosylated prodrugs for a period of up to 48 h after oral administration confirmed their prolonged and delayed pharmacological profile. This was due to the masking of the free carboxylic functions of the NSAIDs. The carboxylic group is crucial for therapeutic effectiveness, meaning that no activity was observed in mice treated with galactosylated prodrugs until the enzymatic pathways cleaved the binding with D-galactose (see Figure 3 and Figure 4).

Although, on the one hand, the carboxylic group is essential for the pharmacological activity of NSAIDs, on the other, it is also partly the cause of their main side effect, i.e., gastrointestinal toxicity. The acute administration of NSAIDs in mice has been observed to cause gastrointestinal lesions of different sizes. Indomethacin showed greater ulcerogenic properties, followed by ketoprofen and finally flurbiprofen and ibuprofen [34,35]. In our work, a slight improvement in the assessment of the damage occurred 48 h after administration, which is when tissue-repair mechanisms were already in place. When we administered the galactosylated prodrugs at the same dosage and evaluated the toxicological profile at the same time point, no damage was observed 4 h after oral administration. This may be attributed to the lack of the ion-trapping phenomenon and thus the lack of irritation of the gastric mucosa resulting from local contact with the carboxyl function.

Forty-eight hours after acute administration, mild ulcerogenic activity was observed in mice treated with the galactosylated analogues. This was probably due to complete hydrolysis of the prodrug, resulting in the release of the active drug which, in turn, led to the blockade of prostaglandin biosynthesis and mucus production in the gastrointestinal tract. It can therefore be stated that, from a toxicological point of view, the galactosylated prodrug strategy was able to alleviate the side effects of NSAIDs.

## 4. Materials and Methods

### 4.1. Chemicals and Materials

The parent drugs (ibuprofen, ketoprofen, flurbiprofen and indomethacin) were purchased from Sigma-Aldrich, Milan, Italy. The galactosylated prodrugs (IbuGAL, OkyGAL, FluGAL and IndoGAL) were synthesized as reported in the work of Magliocca [28]. The plasticware for cultures was obtained from Falcon (Becton Dickinson, Franklin Lakes, NJ, USA). Unless otherwise specified, reagents were obtained from Merck KGaA, (Darmstadt, Germany). 

#### 4.1.1. Lipophilicity

ClogP values were calculated with Bio-Loom for Windows software, v. 1.5, BioByte Corporation, Claremont, CA, USA. The distribution coefficients of the compounds between *n*-octanol and PBS at pH 7.4 (log D^7.4^) were experimentally obtained using the shake-flask technique at room temperature, as previously reported [27]. The concentration of the solutes was measured in the aqueous phase on a Shimadzu UV-2501PC UV spectrophotometer, using calibration curves obtained with standard solutions (r^2^ > 0.99). Each log D value is an average of a minimum of six measurements.

#### 4.1.2. Solubility Assay

Solubility was assessed in deionized water, SGF without pepsin (pH 1.2) and PBS (pH 7.4, 50 mM). The test solid compounds (5 mg) were added to 1 mL of water, PBS and SGF in glass tubes and shaken for 6 h at 25 °C. The suspensions were filtered through a PTFE 0.45 μm filter (VWR), and the resulting solutions were analyzed by means of RP-HPLC (see below). Experiments were performed in triplicate for each compound. Solubility is expressed as mM concentration.

#### 4.1.3. Stability in SGF and PBS

A 10 mM solution of each compound (the galactosylated prodrugs and their parent drugs) was added to either simulated gastric fluid (SGF-without pepsin) or phosphate-buffered saline (PBS, 50 mM). The SGF and PBS buffers were prepared as previously described [27]. The resulting solutions (100 µM) were kept at 37 °C ± 0.5 °C for 48 h; aliquots of 20 µL were withdrawn at appropriate time intervals and analyzed via RP-HPLC (as reported below). The results are expressed as percentages of unmodified galactosylated prodrugs and derived parent drugs after 24 h.

#### 4.1.4. Stability in Human Serum

A solution of each compound (10 mM in DMSO) was added to human serum (sterile-filtered from human male AB plasma, USA origin, sterile-filtered, Sigma-Aldrich) and preheated and maintained at 37 °C ± 0.5 °C. Three-hundred microliters of the resulting solutions (200 µM) was withdrawn at appropriate time intervals and added to 300 µL of CH_3_CN, which contained 0.1% TFA, in order to deproteinize the serum. Samples were sonicated, vortexed and then centrifuged for 10 min at 2150× *g*. Each clear supernatant was filtered using 0.45 µm PTFE filters (VWR International S.r.l., Milano, Italy) and analyzed via RP-HPLC as described below. The results are expressed as percentages of unmodified galactosylated prodrugs and derived parent drugs after 24 h.

#### 4.1.5. Serum Protein Binding

Free- and protein-bound drug separation was obtained via ultrafiltration using commercially available membrane systems (Centrifree ultrafiltration devices with ultracel YM-T membrane, Merck, KGaA, Darmstadt, Germany). A solution of each compound in DMSO was added to human serum (sterile-filtered from human male AB plasma, Sigma-Aldrich) to achieve the 100 μM final concentration with 1% DMSO. One milliliter of the solution was inserted into the sample reservoir of the ultrafiltration device and gently shaken in an orbital shaker for 1 h at 37 °C. The tube was centrifuged at 1000× *g* for 15 min. The concentration of the compound both in the ultrafiltrate and filtrate was analyzed via RP-HPLC under the chromatographic conditions described above and using the calibration curves of standard solutions. The linearity of calibration curves was assessed in a concentration range of 1–100 µM (r^2^ > 0.99). The recovery of the ultrafiltration process was calculated to observe any possible compound loss during the operations in view of the limited solubility of the tested compounds.

Recovery = 100 × [(V_bound_ × C_bound_) + (V_unbound_ × C_unbound_)]/V_initial serum_ × C_initial_

vol._bound_: calculated by dividing the weight of the bound fraction (difference between the weights of the empty sample reservoir and that after ultrafiltration) by its density (0.991 g/mL assessed by weighing five replicates of a known volume of bound fraction).
vol._unbound_: calculated by dividing the weight of the unbound fraction (difference between the weight of the ultrafiltrate cup before and after ultrafiltration) by its density (0.999 g/mL assessed by weighing five replicates of a known volume of unbound fraction).
conc_bound_: calculated using the RP-HPLC method.
conc_unbound_: calculated using the RP-HPLC method (calibration with standard additions).

The average recovery was 98% for all tested compounds.

#### 4.1.6. HPLC Analysis

The reverse-phase HPLC procedure allowed the galactosylated prodrugs and their parent drugs to be separated and quantified. HPLC analyses were performed using a previously used chromatograph system [27]. The analytical column was a ZORBAX Eclipse XDB-C8 (150 × 4.6 mm, 5 µm; Agilent, Santa Clara, CA, USA). The mobile phase consisted of 0.1% aqueous TFA and 0.1% TFA CH_3_CN 50/50 *w*/*w*% at a flow rate of 1.0 mL/min. The injection volume was 20 µL (Rheodyne, Cotati, CA, USA). The column effluent was monitored at 228 nm and 254 nm, referenced against an 800 nm wavelength. The quantitation of compounds was calculated using calibration curves and the linearity of the calibration curves was determined in a concentration range of 1–100 µM (r^2^ > 0.99).

### 4.2. Permeation Measurements across Excised Rat Small Intestine

An evaluation of ex vivo absorption was carried out using permeation measurements in excised rat small intestines, as previously published [38], with some modifications. Briefly, male Wistar rats (200–250 g) were anesthetized with isoflurane, decapitated and exsanguinated. Freshly excised rat duodenum, jejunum and ileum tissue was washed with Krebs–Ringer buffer (KRB) and cut into pieces of 4–5 cm. Then, 0.2 mL of a suspension containing the compounds (5 mg/Kg, PBS + Methocel 3%) were syringed into intestinal sacs and the compound-enriched tissues were incubated in oxygenated KRB (10 mL) at 37 °C with smooth shaking. Each sample solution (0.5 mL) was taken from the serosal side at fixed time intervals up to 120 min and replaced with fresh buffer. Tests were carried out in triplicate for each compound on three intestinal segments from three different rats. The concentration in the incubation buffer was quantified via RP-HPLC, as reported above. The quantitation was performed using calibration curves of all compounds, the linearity of which was determined in a concentration range of 1–100 µM (r^2^ > 0.99).

At the end of the incubation period, the intestinal sacs were opened, washed with fresh buffer, homogenized in ethanol (1 mL, UltraTurrax) and centrifuged at 13,000× *g* for 5 min and the supernatant was dried with nitrogen. After resolubilization in 0.5 mL of methanol, the concentrations of the galactosylated prodrugs and prodrug-derived parent drugs were quantified using RP-HPLC.

### 4.3. Animals

Adult male CD1 mice weighing 25–35 g, purchased from Charles River (Calco, Italy), were used for in vivo and ulcerogenicity studies. Male Wistar rats weighing 200–250 g, purchased from Charles River (Italy), were used for ex vivo absorption evaluations according to [38]. The animals were kept under standard environmental conditions of controlled temperature (22  °C ±  2 °C) and humidity with a 12/12 h light/dark cycle. Food and water were supplied ad libitum, except before the experimental days, when the animals fasted for 12 h. All experiments were approved by the Italian Ministry of Health and were in accordance with the guidelines for the use of laboratory animals. Our best efforts were made to reduce the number of animals used and to minimize their suffering.

#### 4.3.1. Carrageenan-Induced Hyperalgesia

Carrageenan-induced paw edema was induced by injecting λ-carrageenan (1% *w*/*v* in sterile water, 20 μL) subcutaneously into the hind paws of mice using a 27 gauge needle. The drugs were administered orally by gavage 1 h earlier, and the mice were tested 2, 4, 6, 24, 48, 72 and 96 h after the λ-carrageenan injection. As previously reported [39], mouse paw edema developed in two distinct phases: an acute first phase peaking at 4 h and a second phase peaking at 72 h after carrageenan challenge. The increase in paw volume was evaluated using a plethysmometer (Ugo Basile, Milan, Italy) and reported as the difference between the paw volume measured at each time point and the basal paw volume measured immediately before carrageenan injection.

#### 4.3.2. Acetic Acid Writhing Test

The acetic-acid-induced writhing test was performed as reported previously [40]. Briefly, the drugs were orally administered to mice 1 h before the intraperitoneal injection of 0.6% acetic acid (10 mL/kg). The number of abdominal constrictions and extensions of the trunk and hind limbs were counted for each group of mice, starting from 5 min after the injection of acetic acid up to 20 min afterwards, and expressed as the number of writhing episodes.

#### 4.3.3. Ulcerogenicity Studies

NSAID-induced gastric damage in mice was evaluated following the procedure described by Chan et al. [41]. Solutions of either the free-acid parent drug (10 mg/kg) or equimolecular amounts of the galactosylated prodrug or vehicle alone (CMC, 0.5%) were administered orally in fasted (16–18 h) mice (n = 6 per group). Mice were euthanized either 4 h or 48 h after treatment, and the stomach was excised along its greater curvature and rinsed with normal saline. The mucosa was then examined by means of a magnifying glass for the presence of irritation or frank hemorrhagic lesions (ulcers). Irritation was assigned a score of 0, and ulcerations were scored according to their length (a score of 1 for lesions with a length between 1 and 2 mm, a score of 2 for lesions with a length between 2 and 3 mm, a score of 3 for lesions greater than 3 mm). The sum of total scores was used for comparison.

### 4.4. Statistical Analysis

All analyses were conducted using Graph-Pad Prism (GraphPad Prism, version 7.0 from GraphPad Software Inc., San Diego, CA, USA). For the in vivo antinociceptive tests, the significance of differences between groups was determined using two-way analysis of variance (ANOVA), followed by a Turkey’s post hoc test for multiple comparisons. The level of statistical significance was * *p* < 0.05 and was evaluated vs. the control group.

## 5. Conclusions

By conjugating ibuprofen, ketoprofen, flurbiprofen and indomethacin to D-galactose, the physicochemical properties and bioproperties of their respective parent drugs were changed in such a way as to prolong their pharmacological activity and reduce their toxicity. If a NSAID candidate suffers from unmet requirements, its galactosylated analogue may satisfy them. In light of the results obtained in the present work and previously with ketorolac, aceclofenac and paracetamol, the galactosylated prodrug strategy can be considered a problem-solving technique that can be adopted to overcome the drawbacks of NSAIDs.

## Figures and Tables

**Table 1:** Physicochemical characterization of galactosylated prodrugs and parent drugs: lipophilicity, calculated as log P (ClogP); experimental distribution coefficient at pH 7.4 (log D^7.4^); and solubility (mM) in water, SGF (pH 1.2) and PBS (pH 7.4).

Compound	Lipophilicity		Solubility at 25 °C (mM) ^c^		
	ClogP ^a^	logD^7.4 b^	Water	SGF pH 1.2	PBS pH 7.4
IbuGAL	1.92	1.73 ± 0.04	1.94	2.53	2.93
Ibuprofen	3.68	0.82 ± 0.04	0.22	0.58	23.5
OkyGAL	1.00	0.92 ± 0.01	23.36	8.34	5.53
Ketoprofen	2.76	−0.22 ± 0.03	0.46	0.21	7.71
FluGAL	1.99	1.89 ± 0.09	5.21	1.44	1.01
Flurbiprofen	3.75	0.81 ± 0.01	0.07	0.02	5.43
IndoGAL	2.42	2.05 ± 0.06	0.07	0.08	0.13
Indomethacin	4.18	0.91 ± 0.01	0.07	<LOQ ^d^	2.25

^a^ Calculated using Bio-Loom for Windows, v. 1.5, BioByte Corporation, Claremont, CA, USA. ^b^ Results are expressed as mean values ± SD. ^c^ Results are expressed as mean values, SD < 0.01. ^d^ LOQ (limit of quantitation, HPLC) = 1 µM.

**Table 2:** The chemical stability in SGF (pH 1.2) and PBS (pH 7.4), enzymatic stability in human serum and serum protein binding (%bound) of galactosylated prodrugs compared to relative NSAIDs.

Compound	Stability at 37 °C % Compounds after 24 h of Incubation (Mean ± SD)			Serum Protein Binding % Bound ^a^
	SGF pH 1.2 ^a^	PBS pH 7.4 ^a^	Human Serum ^a^	
IbuGAL	98.3 ± 1.0	81.2 ± 2.5	54.1 ± 2.2	97.2 ± 0.4
Ibuprofen (IbuGAL)	3.4 ± 1.1	9.4 ± 1.5	40.8 ± 6.2	
Ibuprofen	stable	stable	stable	99.98 ± 0.01
OkyGAL	97.9 ± 0.7	91.0 ± 0.2	63.0 ± 8.7	95.0 ± 1.6
Ketoprofen (OkyGAL)	1.4 ± 0.5	9.6 ± 0.8	30.4 ± 7.7	
Ketoprofen	stable	stable	stable	99.98 ± 0.01
FluGAL	98.9 ± 0.1	87.3 ± 2.1	57.3 ± 1.4	96.1 ± 0.5
Flurbiprofen (FluGAL)	1.1 ± 0.4	11.6 ± 0.8	44.5 ± 2.4	
Flurbiprofen	stable	stable	stable	99.98 ± 0.01
IndoGAL	97.8 ± 0.4	89.0 ± 1.6	82.3 ± 2.2	99.9 ± 0.1
Indomethacin (IndoGAL)	2.6 ± 0.5	11.5 ± 1.5	17.8 ± 4.6	
Indomethacin	stable	stable	stable	99.98 ± 0.01

^a^ Results are expressed as mean values ± SD.

**Table 3:** Ex vivo fraction absorbed (F_a_) of galactosylated prodrugs compared to relative NSAIDs.

Compound	Fraction Absorbed (F_a_) ± SE		
	Duodenum	Jejunum	Ileum
IbuGAL	0.013 ± 0.006	0.026 ± 0.014	0.031 ± 0.016
Ibuprofen (IbuGAL)	0.043 ± 0.015	0.028 ± 0.011	0.036 ± 0.011
Ibuprofen	0.94 ± 0.2	0.84 ± 0.11	0.98 ± 0.10
OkyGAL	0.038 ± 0.012	0.015 ± 0.012	0.018 ± 0.006
Ketoprofen (OkyGAL)	0.10 ± 0.004	0.060 ± 0.018	0.076 ± 0.007
Ketoprofen	0.53 ± 0.02	0.48 ± 0.03	0.46 ± 0.02
FluGAL	0.060 ± 0.014	0.031 ± 0.015	0.033 ± 0.008
Flurbiprofen (FluGAL)	0.13 ± 0.01	0.075 ± 0.023	0.096 ± 0.009
Flurbiprofen	0.69 ± 0.02	0.62 ± 0.04	0.57 ± 0.04
IndoGAL	Not Evaluable	Not Evaluable	Not Evaluable
Indomethacin (IndoGAL)			
Indomethacin	0.040 ± 0.005	0.047 ± 0.012	0.094 ± 0.017

**Table 4:** Ulcerogenic activity of parent drugs and galactosylated prodrugs 4 and 48 h after treatment.

Compounds	Oral Dose (mg/kg)	Gastric Lesion Score (4 h) ^a^	Gastric Lesion Score (48 h) ^a^
Control (CMC 0.5%)	-	0	0
Ibuprofen	10	2 **^##^**	1.5
IbuGAL	17.8	0 **	1
Ketoprofen	10	2.5 **^###^**	1.5
OkyGAL	16.4	0 ****	1
Flurbiprofen	10	2 **^##^**	1.5
FluGAL	16.7	0 **	0.5
Indomethacin	10	3 **^####^**	2 **^##^**
IndoGAL	14.5	0 ****	1

^a^ A score of 0 was assigned for irritation, a score of 1 for lesions with a length between 1 and 2 mm, a score of 2 for lesions with a length between 2 and 3 mm and a score of 3 for lesions greater than 3 mm. Significance of galactosylated prodrug versus respective parent drug: **** *p* < 0.0001, ** *p* < 0.01. Significance of parent drug versus control: ^####^ *p* < 0.0001, ^###^ *p* < 0.001, ^##^ *p* < 0.01.

## Data Availability

Data is contained within the article.
